# Immediate and long-term brain activation of acupuncture on ischemic stroke patients: an ALE meta-analysis of fMRI studies

**DOI:** 10.3389/fnins.2024.1392002

**Published:** 2024-07-19

**Authors:** Yuan Zhang, Hai Lu, Xuesong Ren, Junfeng Zhang, Yu Wang, Chunhong Zhang, Xiaofeng Zhao

**Affiliations:** ^1^Department of Acupuncture and Moxibustion, First Teaching Hospital of Tianjin University of Traditional Chinese Medicine, Tianjin, China; ^2^Graduate College, Tianjin University of Traditional Chinese Medicine, Tianjin, China; ^3^National Clinical Research Center for Chinese Medicine Acupuncture and Moxibustion, Tianjin, China; ^4^Department of Traditional Chinese Medicine, Nanjing Drum Tower Hospital, Affiliated Hospital of Medical School, Nanjing University, Nanjing, China; ^5^Department of Rehabilitation, Second Teaching Hospital of Tianjin University of Traditional Chinese Medicine, Tianjin, China; ^6^Department of Acupuncture and Moxibustion, Baoan Pure Traditional Chinese Medicine Treatment Hospital, Shenzhen, China

**Keywords:** ischemic stroke, acupuncture, functional MRI, activation likelihood estimation, meta analysis

## Abstract

**Background:**

Acupuncture, as an alternative and complementary therapy recommended by the World Health Organization for stroke treatment, holds potential in ameliorating neurofunctional deficits induced by ischemic stroke (IS). Understanding the immediate and long-term effects of acupuncture and their interrelation would contribute to a better comprehension of the mechanisms underlying acupuncture efficacy.

**Methods:**

Activation likelihood estimation (ALE) meta-analysis was used to analyze the brain activation patterns reported in 21 relevant functional neuroimaging studies. Among these studies, 12 focused on the immediate brain activation and 9 on the long-term activation. Single dataset analysis were employed to identify both immediate and long-term brain activation of acupuncture treatment in IS patients, while contrast and conjunction analysis were utilized to explore distinctions and connections between the two.

**Results:**

According to the ALE analysis, immediately after acupuncture treatment, IS patients exhibited an enhanced cluster centered around the right precuneus (PCUN) and a reduced cluster centered on the left middle frontal gyrus (MFG). After long-term acupuncture treatment, IS patients showed an enhanced cluster in the left PCUN, along with two reduced clusters in the right insula (INS) and hippocampus (HIP), respectively. Additionally, in comparison to long-term acupuncture treatment, the right angular gyrus (ANG) demonstrated higher ALE scores immediately after acupuncture, whereas long-term acupuncture resulted in higher scores in the left superior parietal gyrus (SPG). The intersecting cluster activated by both of them was located in the left cuneus (CUN).

**Conclusion:**

The findings provide initial insights into both the immediate and long-term brain activation patterns of acupuncture treatment for IS, as well as the intricate interplay between them. Both immediate and long-term acupuncture treatments showed distinct patterns of brain activation, with the left CUN emerging as a crucial regulatory region in their association.

**Systematic Review Registration:**

https://www.crd.york.ac.uk/prospero/, CRD42023480834.

## 1 Introduction

Ischemic stroke (IS), a critical subtype of stroke, stands out as a primary neurovascular factor contributing significantly to both mortality and disability (Feske, 2021). It is distinguished by specific disruptions in the blood circulation of the brain, leading to tissue damage and neurological impairment. Current statistics indicate that globally, over 77 million individuals have experienced IS, with an annual increase of approximately 7.6 million new cases (Feigin et al., 2022). Alarmingly, this number continues to rise, and considering the substantial long-term risk of recurrence, all these factors impose an immense burden on both global healthcare systems and societies at large (Ding et al., 2022; Fan et al., 2023). The current treatment approaches for IS encompass vascular reperfusion therapies, such as intravenous thrombolysis or mechanical thrombectomy, secondary prevention strategies involving antiplatelet drugs, anticoagulants, and statins, along with post-stroke care and rehabilitation interventions (Herpich and Rincon, 2020; Hasan et al., 2021). Despite notable progress in reducing the occurrence of stroke-related complications and mortality rates, certain limitations persist. For instance, the treatment window for thrombolytic drugs is relatively narrow and strictly regulated (Saini et al., 2021). Additionally, factors such as age and comorbidities significantly contribute to variations in rehabilitation outcomes (Stinear et al., 2020). Overcoming these limitations and exploring targeted prevention and intervention strategies is necessary for advancing IS research, as well as the pursuit of innovative therapies.

Acupuncture is a traditional Chinese medicine technique that regulates the flow of qi and restores the balance of yin and yang by inserting fine needles into specific acupoints on the human body, and is characterized by its simplicity, convenience, and cost-effectiveness (Kaptchuk, 2002). For 1,000 of years, acupuncture has been used to treat various diseases and disorders, including stroke (Huang et al., 2021). Both clinical and experimental evidence suggest that acupuncture holds significant potential in improving neurological deficits induced by IS, especially for the sequelae of stroke, making acupuncture an increasingly promising intervention for stroke patients (Zhang et al., 2014; Yang et al., 2016; Zhong et al., 2022). However, the mechanism of acupuncture in improving IS is not clear yet. The modulation of neuroplasticity by acupuncture may be the key factor for its effectiveness (Chavez et al., 2017). Current researches show that acupuncture can modulate neuroplasticity in the central nervous system (CNS) by altering neural structure and function (Zhang et al., 2021). This neuroplasticity manifests as immediate functional changes and prolonged structural and functional alterations. During acupuncture, the body undergoes acute physiological responses, which may, in turn, stimulate processes such as neural generation, synaptic sprouting, and regeneration. These processes, in combination, promote the growth of new neurons and facilitate the brain’s self-reorganization through the formation of new neural connections, then lead to lasting changes in the neural circuits related to sensation, movement, cognition, and more (Qin et al., 2022; Mu et al., 2023).

However, previous meta-analyses have primarily focused on the long-term effects of acupuncture on IS, investigating alterations in brain activity or connectivity following regular acupuncture sessions. Interestingly, in our clinical practice, we have observed immediate symptom improvement in patients following acupuncture, a viewpoint supported by some studies (Du et al., 2016; Wang H. Q. et al., 2018). The immediate effect of acupuncture may play an important role in its efficacy, with its correlation to long-term effectiveness extending beyond cumulative effects. Currently, there is limited research in these aspects. A comprehensive understanding of both immediate and long-term effects of acupuncture, and their interaction, is essential for a better comprehension of the mechanisms underlying acupuncture efficacy. This knowledge is instrumental in optimizing acupuncture interventions in clinical settings and tailoring treatment plans to address diverse aspects of post-stroke recovery.

Functional magnetic resonance imaging (fMRI) is a non-invasive technique that measures changes in blood oxygen level-dependent (BOLD) signals within the brain (Logothetis, 2008). As a primary tool in human neuroimaging research, fMRI offers a unique opportunity to reveal the neural processes underlying acupuncture modulation of brain regions and networks, thereby expanding our comprehension of the neural mechanisms involved in brain reorganization post-acupuncture (He et al., 2015). However, the heterogeneity of fMRI research results is a common challenge due to factors such as a limited number of subjects and variations in experimental procedures. To precisely elucidate the cerebral responses of acupuncture treatment for IS, it is imperative to integrate existing studies and conduct a comprehensive analysis of their results. Among the various techniques available, activation likelihood estimation (ALE) stands out as the most commonly used coordinate-based meta-analysis approach in neuroimaging data synthesis (Wager et al., 2007). ALE treats reported activation foci from different studies as spatial probability distributions centered around specific coordinates (Eickhoff et al., 2012). By calculating the joint probability of activation at each voxel, ALE maps integrate information about the location of brain activation across diverse studies (Eickhoff et al., 2012). In this study, we employed the ALE method to perform an integrative analysis of fMRI studies on acupuncture treatment for IS, aiming to obtain more reliable results than those found in single-study reports.

We have gathered previous fMRI studies on acupuncture treatment for IS and extracted the reported coordinate information. Specifically, we have divided the coordinates into two datasets, one dataset containing activated coordinates of brain regions immediately after acupuncture in IS patients, and the other containing activated coordinates after acupuncture treatment for one or several courses. By performing a series of ALE meta-analyses on the two datasets, we aim to further explore the immediate and long-term brain activation patterns of acupuncture treatment for IS and investigate the relationship between them. This exploration aims to provide novel insights into the neural mechanisms of acupuncture for IS from a macro perspective, thereby offering valuable considerations for designing and optimizing acupuncture treatment plans for future patients.

## 2 Methods

This meta-analysis followed the guidelines outlined in the Preferred Reporting Items for Systematic Reviews and Meta-Analysis of Acupuncture (PRISMA-A) (Wang et al., 2019) recommended checklist to ensure a comprehensive and transparent assessment, and it has been duly registered in PROSPERO (No. CRD42023480834).

### 2.1 Literature search and study selection

The articles were sourced from four English bibliographic databases: PubMed,^1^ The Cochrane Library,^2^ Embase,^3^ and Web of Science,^4^ as well as four Chinese bibliographic databases: China National Knowledge Infrastructure (CNKI),^5^ Chinese Biomedical Literature Database (CBM),^6^ China Science and Technology Journal Database (VIP),^7^ and Wanfang Data Knowledge Service Platform Database (WF).^8^ The search covered the inception of these databases until December 1st, 2023, with language restricted to English and Chinese. "fMRI," "ischemic stroke," and "acupuncture" were utilized as free terms and subject terms in various combinations within the search strategy. Refer to the Supplementary materials for a detailed search strategy. Additionally, a meticulous examination of the references in these articles was undertaken to ensure that no relevant literature was omitted.

The articles obtained through the search were screened based on inclusion and exclusion criteria. The inclusion criteria were as follows: 1. The included patients met the diagnostic criteria for IS; 2. Utilized whole-brain voxel analysis with fMRI and reported activation peak coordinates in standard MNI or Talairach coordinate format; 3. Immediate effects of acupuncture should be examined through single-needle task-based studies, while long-term effects should be investigated through resting-state studies [including regional homogeneity (ReHo), amplitude of low frequency fluctuation (ALFF)/fractional amplitude of low frequency fluctuation (fALFF), and functional connectivity (FC) analysis with the primary sensory-motor cortex of the affected side as the regions of interest (ROI)], with treatment allowing combination with basic drug and rehabilitation; 4. Conducted statistical comparison of pre-and post-acupuncture brain effects in patients. Exclusion criteria were as follows: 1. Non-clinical research, such as reviews, meta-analyses, animal experiments, and protocols; 2. Acupuncture groups with fewer than 5 subjects; 3. Articles that did not report coordinate data or reported incomplete coordinate information, with unsuccessful attempts to contact the authors; 4. Duplicated publications or literature with repeated data. Two researchers (YZ and YW) independently searched and screened the literature according to the predefined search strategy and eligibility criteria, and cross-checked their results after completion. Any discrepancies were resolved by discussion.

### 2.2 Quality assessment and data extraction

Since there is no standard checklist for the quality assessment of functional neuroimaging studies, we divided the included literature into randomized controlled trials (RCTs) and non-RCTs, and used the Cochrane risk of bias (ROB) tools (Sterne et al., 2019)^9^ and MINORS scale (Slim et al., 2003) to evaluate their methodological quality, respectively. In addition, considering the specificity of functional imaging examination, a checklist published in a previous meta-analysis was employed to score the completeness of the articles (Ha et al., 2022). The checklist contained two categories: sample characteristics and methodology and reporting, totaling 10 items with a maximum score of 10 points (Supplementary Table S5). Literature with a score greater than 6 points was considered eligible for subsequent data extraction and analysis.

Next, we extracted data from the included literature by reading the full texts. Since this study mainly explored the immediate and long-term brain activation of acupuncture, the extracted data mainly focused on the information of the acupuncture group that met the inclusion and exclusion criteria, including the publication details (title, first author, publishing year), the baseline data of the subjects in the acupuncture group (sample size, gender, age, disease duration), the trial design (study type, acupuncture method, MRI parameters) and the outcomes (peak coordinates, t/z values, cluster sizes and threshold). If a single article involved two different acupuncture interventions (excluding sham acupuncture), data extraction was conducted for each intervention separately. Similarly, if the same article employed two different analysis methods that met the inclusion criteria, the data extraction was carried out for each method independently. Quality assessment and data extraction were performed by two reviewers (HL and JFZ), and discrepancies were resolved by another senior researcher (XFZ).

### 2.3 Activation likelihood estimation

In this study, we used Ginger ALE 3.0.2^10^ (Eickhoff et al., 2009, 2012; Turkeltaub et al., 2012) to perform ALE meta-analysis on the neuroimaging data. Specifically, we conducted the single dataset analysis for immediate and long-term post-acupuncture brain activation patterns on individual datasets separately. Subsequently, a contrast analysis was performed on both datasets to further examine similarities and differences between the immediate and long-term brain activation patterns. The specific operation steps are as follows.

Firstly, we manually inputted the foci data from the included literature into standard format text files according to the user manual, and used the “icbm2tal” tool (Lancaster et al., 2007; Laird et al., 2010) to convert the Talairach space coordinates to MNI coordinates. The ALE calculations involve generating 3D Modeled Activation (MA) maps for foci groups, using masks, foci, and Gaussian blur with FWHM derived from subject size (Eickhoff et al., 2009). Ginger ALE utilizes histograms of MA map values to determine the null distribution, producing a table of *p* values for ALE scores, and creating 3D *p*-value images accordingly (Eickhoff et al., 2012).

In the single dataset analysis, we converted the foci data of articles focusing on immediate and long-term changes in brain modulation into two separate datasets. These datasets were then input into the Ginger ALE, with MNI152 chosen as the coordinate space and Cluster-level FWE correction at *p* < 0.01. A Monte-Carlo simulation with 1,000 iterations was conducted to calculate the *p*-values, and the statistical significance threshold was set at *p* < 0.05 (Muller et al., 2018).

Contrast analysis compared the above datasets. Two ALE contrast images (Immediate activation > Long-term activation and Long-term activation > Immediate activation) were created by subtracting one input image from another. Additionally, a conjunction image (Immediate activation ∩ Long-term activation) was generated by using the voxel-wise minimum values from the input ALE images. To address differences in study sizes, simulated data was generated by pooling and randomly dividing foci datasets into two groups that matched the original sizes (Eickhoff et al., 2011). We first merged the foci data of immediate and long-term activation into a pooled text file. Following the analysis method used for the single datasets, the ALE image of the pooled text file was generated. We then inputted the three ALE images (immediate activation, long-term activation, and pooled activation) into Ginger ALE for contrast analysis, with parameter settings at *p* < 0.01 with 1,000 permutations.

Finally, in the MatLab R2017b software environment, the acquired ALE images were processed and visualized using the BrainNet Viewer Version 1.7^11^ (Xia et al., 2013), a toolbox for brain imaging data processing and analysis.

## 3 Results

### 3.1 Search results

**Figure 1:**
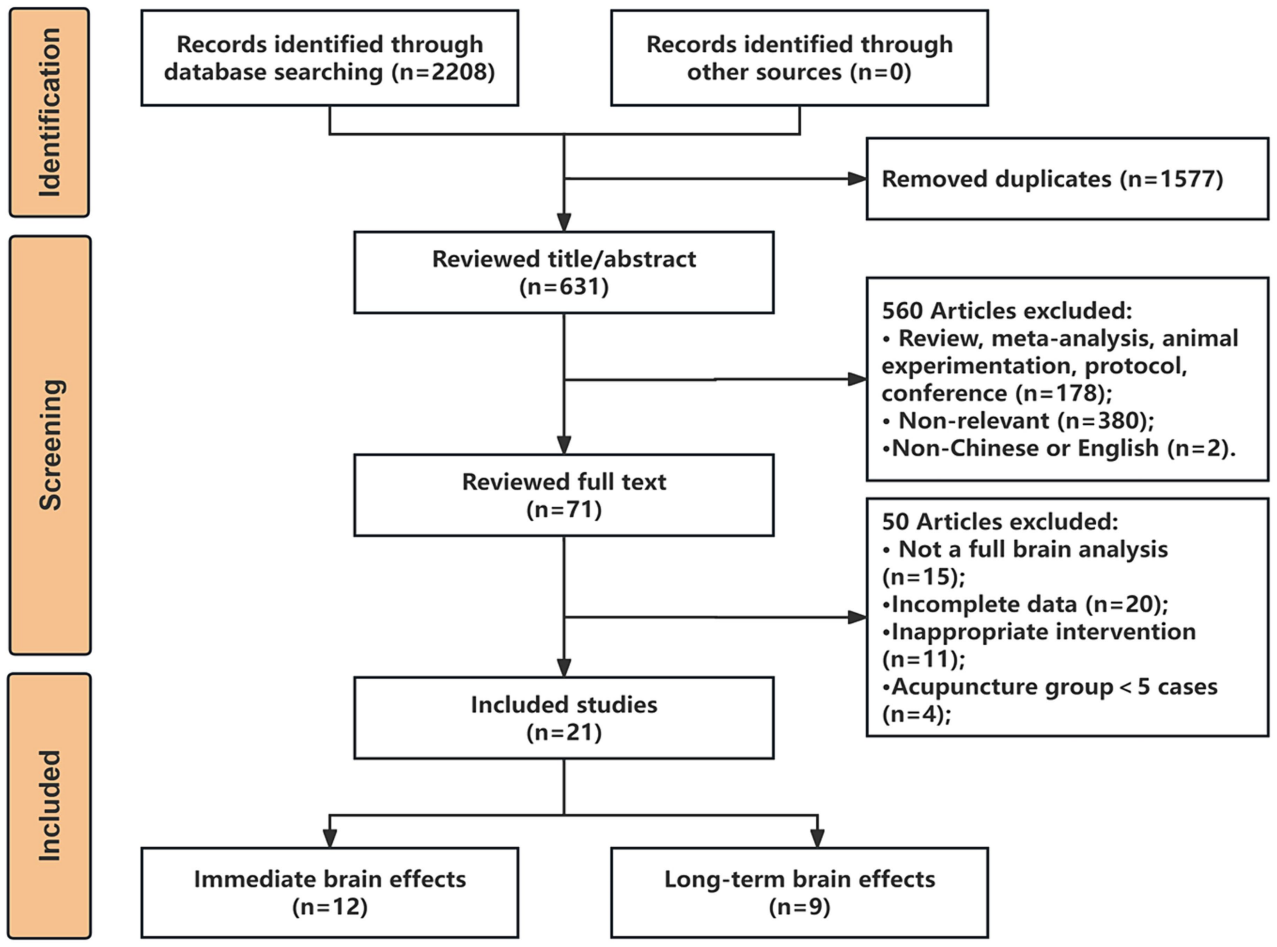
The flow diagram of the literature search and study selection.

A total of 2,208 articles were retrieved and imported into Endnote X9 software for literature management, and after a rigorous selection process, as illustrated in Figure 1, we finally included 21 studies that met the criteria for the analysis, comprising 12 articles on the immediate brain modulation of acupuncture and 9 articles on the long-term modulation of acupuncture.

All included articles underwent evaluations for both content completeness and quality. The content completeness scores for all 21 articles were above 6 points. 9 RCT studies used Cochrane ROB tools for quality evaluation, while 12 non-RCTs used the MINORS scale. The results of content completeness evaluation and quality assessment can be found in the Supplementary materials.

Additionally, through data extraction from the literature, we obtained two foci datasets on the immediate and long-term brain activation of acupuncture in IS patients. The immediate activation dataset, derived from 12 articles with 13 contrasts, included 127 IS patients, with 100 up foci (post-acupuncture > pre-acupuncture) and 70 down foci (post-acupuncture < pre-acupuncture). The long-term activation dataset, from 9 articles with 10 contrasts, included 162 IS patients, with 54 up foci and 19 down foci. Detailed information about the articles is provided in Tables 1, 2.

**Table 1:** Overview of the included studies on immediate brain activation.

Number	Study	Sample size (n)	Gender (M/F)	Age (mean ± SD)	Duration (mean ± SD)	Acupoint	Acupuncture method	Magnetic field	Coordinate	Foci (n)	Threshold
1	Dong et al. (2010)	5	4/1	NA	NA	GB34	MA	1.5 T	Talairach	up: 13	*p* < 0.05 cor
										down: 2	
2	Chen et al. (2011)	6	5/1	58.33 ± 5.65	7.50 ± 5.47	TE5	MA	3.0 T	MNI	up: 21	*p* < 0.001 cor
										down: 6	
3	Zheng et al. (2012)	6	5/1	56.33 ± 9.52	3.83 ± 2.93	TE5	MA	3.0 T	MNI	up: 16	*p* < 0.001 cor
										down: 20	
4	Xiao et al. (2012)	6	4/2	55.67 ± 8.41	6.08 ± 6.40	TE5	MA	3.0 T	MNI	down: 3	*p* < 0.001 cor
5	Cho et al. (2013)	11	7/4	57.90 ± 6.49	3.39 ± 0.60	LI11	MA	3.0 T	Talairach	up: 1	*P* < 0.0005 cor
						ST36				up: 4	
6	Huang et al. (2013)	10	9/1	56.10 ± 5.53	5.30 ± 3.71	TE5	MA	3.0 T	Talairach	up: 10	*p* < 0.001 cor
										down: 10	
7	Qi et al. (2014)	8	7/1	55.00 ± 5.63	4.63 ± 3.85	TE5	MA	3.0 T	Talairach	up: 8	*p* ≤ 0.001 cor
										down: 10	
8	Li et al. (2015)	5	4/1	56.80 ± 5.02	5.20 ± 3.71	TE5	MA	3.0 T	Talairach	up: 1	*p* ≤ 0.05 cor
										down: 2	
9	Wang et al. (2016)	20	14/6	58.60 ± 8.12	NA	LI4, TE5	EA	3.0 T	MNI	up: 18	*p* < 0.001 cor
										down: 4	
10	Zhang et al. (2017)	20	10/10	52.8 ± 8.2	2.2 ± 1.2	TE5	MA	3.0 T	Talairach	down: 10	*p* ≤ 0.001 cor
11	Fu et al. (2019)	20	13/7	61.32 ± 8.53	NA	GB34	MA	3.0 T	Talairach	up: 6	*p* < 0.05 cor
12	Chen et al. (2020)	10	7/3	57.70 ± 7.69	24.60 ± 9.14	LI11, ST36	MA	3.0 T	MNI	up: 2	*p* < 0.05 cor
										down: 3	

M, male; F, female; SD, standard deviation; NA, not available; MA, manual acupuncture; EA, electroacupuncture; NRER, non-repetitive event-related; MNI, montreal neurological institute.

**Table 2:** Overview of the included studies on long-term brain activation.

Number	Study	Sample size (n)	Gender (M/F)	Age (mean ± SD)	Duration (mean ± SD)	Acupuncture method	Duration of treatment	Magnetic field	Coordinate	Foci (n)	Threshold
1	Xie et al. (2013)	9	5/4	65.50 ± 2.32	NA	MA + Conv	20 in 4 weeks	3.0 T	Talairach	up: 12	*p* < 0.05 cor
										down: 12	
2	Wu et al. (2017)	11	7/4	69.36 ± 4.14	52.82 ± 15.55	MA + Conv	20 in 4 weeks	3.0 T	Talairach	up: 8	*p* < 0.05 cor
3	Ye and Guo (2018)	42	22/20	55.67 ± 8.38	NA	MA + Conv	14 in 2 weeks	1.5 T	MNI	up: 3	*p* < 0.05 cor
										down: 2	
4	Liu et al. (2020)	6	4/2	59.67 ± 2.50	NA	MA + Conv	12 in 1 week	3.0 T	MNI	up: 15	*p* < 0.05 cor
5	Fan et al. (2021)	18	9/9	63.91 ± 3.27	24.95 ± 3.19	EA + Conv	24 in 4 weeks	3.0 T	MNI	up: 4	*p* < 0.05 cor
										down: 3	
6	Liu H. et al. (2021)	15	12/3	56.47 ± 8.25	NA	MA + Conv	12 in 1 week	3.0 T	MNI	up: 2	*p* < 0.05 cor
										up: 1	
7	Liu Y. et al. (2021)	17	4/13	57.76 ± 6.02	11.53 ± 2.27	MA + Conv	20 in 4 weeks	3.0 T	MNI	up: 2	*p* < 0.05 cor
8	Yi et al. (2021)	21	13/8	66.2 ± 9.17	NA	EA + Conv	28 in 4 weeks	3.0 T	MNI	up: 2	*p* < 0.005 cor
										down: 2	
9	Peng et al. (2023)	23	17/6	57.67 ± 7.13	NA	MA + Conv	12 in 4 weeks	3.0 T	MNI	up: 5	*p* < 0.05 cor

M, male; F, female; SD, standard deviation; NA, not available; MA, manual acupuncture; EA, electroacupuncture; Conv, conventional treatment; MNI, montreal neurological institute.

### 3.2 ALE results

#### 3.2.1 Immediate brain activation

**Figure 2:**
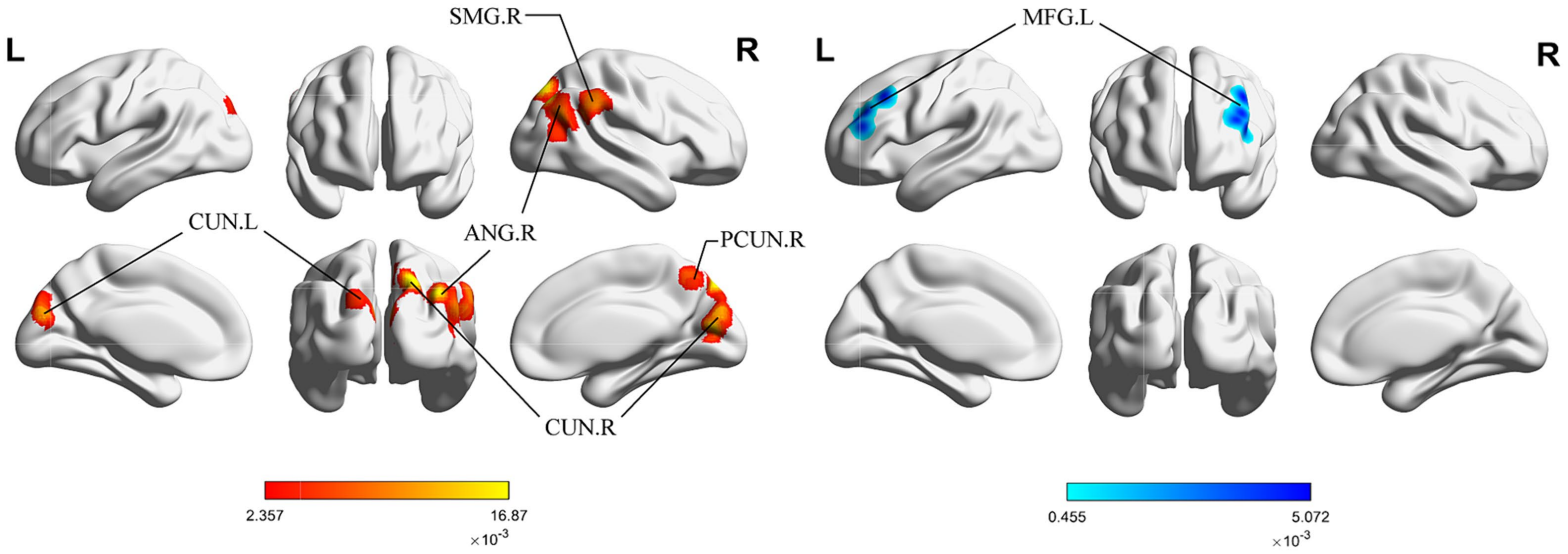
ALE map for immediate activation of acupuncture. The color bar represents the ALE scores, with enhanced clusters depicted in red and reduced clusters in blue (*p* < 0.05, cluster-level FWE). PCUN, precuneus; CUN, cuneus; ANG, angular gyrus; SMG, supramarginal gyrus; MFG, middle frontal gyrus; R, right; L left.

Figure 2 shows the immediate post-acupuncture changes in brain activation of patients with IS, based on the single dataset analysis. It includes a significantly enhanced cluster and a markedly reduced cluster. The center of the enhanced cluster is located at the precuneus (PCUN) of the right superior parietal lobule (x, y, z = 20, −78, 48), which contains parts of the cuneus (CUN), middle temporal gyrus (MTG), superior temporal gyrus (STG), supramarginal gyrus (SMG), and angular gyrus (ANG). The center of the reduced cluster is located at the middle frontal gyrus (MFG) of the left hemisphere (x, y, z = −40, 36.5, 28.5). The detailed information for these clusters is listed in Table 3.

**Table 3:** Immediate activation likelihood clusters after acupuncture.

Cluster	Cluster size(mm^3^)	Peak MNI coordinates			ALE	Z	Side	Anatomical regions	BA
		x	y	z					
Post > Pre									
1	27,152	20	−78	48	0.01687049	5.3095183	R	Precuneus	19
		36	−74	40	0.016535562	5.243561	R	Precuneus	19
		0	−82	26	0.016485503	5.2335014	L	Cuneus	18
		4	−76	14	0.013915152	4.6875906	R	Cuneus	23
		54	−68	26	0.011794807	4.204568	R	Middle Temporal Gyrus	39
		58	−54	24	0.01085433	3.9878206	R	Superior Temporal Gyrus	39
		60	−42	36	0.009889567	3.768477	R	Supramarginal Gyrus	40
		12	−64	48	0.008159517	3.353732	R	Precuneus	7
		8	−82	40	0.005137592	2.4657152	R	Cuneus	19
		39	−71	40	0.005109134	2.4618075	R	Angular Gyrus	19
		−18	−86	34	0.005072881	2.4510827	L	Cuneus	18
Post < Pre									
1	13,208	−40	36.5	28.5	0.00421329	2.7776585	L	Middle Frontal Gyrus	9

MNI, montreal neurological institute; ALE, activation likelihood estimation; BA, brodmann area; R, right; L left.

#### 3.2.2 Long-term brain activation

**Figure 3:**
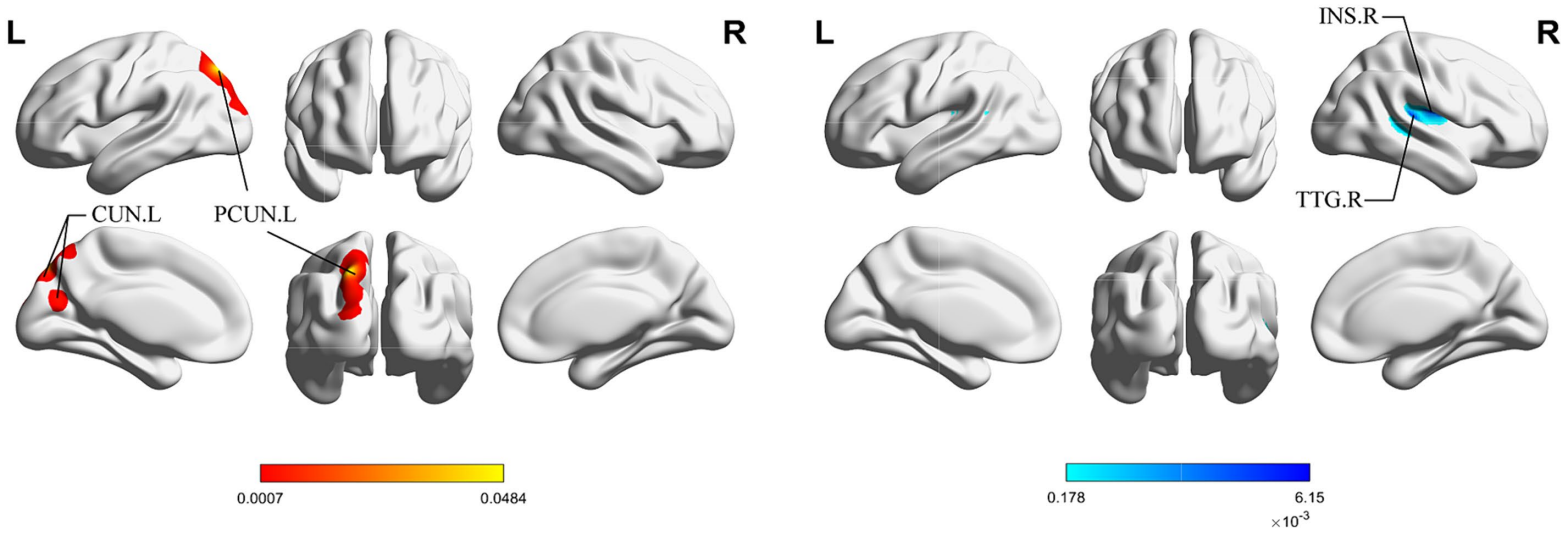
ALE map for long-term activation of acupuncture. The color bar represents the ALE scores, with enhanced clusters depicted in red and reduced clusters in blue (*p* < 0.05, cluster-level FWE). PCUN, precuneus; CUN, cuneus; INS, insula; TTG, transverse temporal gyrus; R, right; L left.

Figure 3 shows the long-term acupuncture changes in brain activation of patients with IS, including one cluster of significant enhancement and two clusters of significant reduction. The center of the enhanced cluster is located in the left PCUN (x, y, z = −20, −78, 48), and also contains parts of the middle occipital gyrus (MOG) and the CUN. Among the reduced clusters, the first cluster has its center in the right insula (INS) (x, y, z = 42, −15, 20), and also contains parts of the transverse temporal gyrus (TTG). The center of the second cluster is found in the left hippocampus (HIP) within the limbic system (x, y, z = −35, −47, 12), involving a segment of the caudate tail (CNT). The detailed information for these clusters is listed in Table 4.

**Table 4:** Long-term activation likelihood clusters after acupuncture.

Cluster	Cluster size (mm^3^)	Peak MNI coordinates			ALE	Z	Side	Anatomical regions	BA
		x	y	z					
Post > Pre									
1	22,488	−20	−78	48	0.04838178	10.42933	L	Precuneus	19
		−19	−90	18	0.0049557	2.698074	L	Middle Occipital Gyrus	18
		−16	−64	54	0.004918353	2.676043	L	Precuneus	7
		−20	−90	34	0.00484734	2.6571395	L	Cuneus	19
		−18	−74	22	0.004842125	2.6514213	L	Precuneus	31
		−12	−73	21	0.004842124	2.6514213	L	Cuneus	18
Post < Pre									
1	12,816	42	−15	20	0.006149536	3.294011	R	Insula	13
		43	−34	9	0.005986688	3.158178	R	Transverse Temporal Gyrus	41
2	11,424	−35	−47	12	0.005986688	3.158178	L	Hippocampus	/
		−25	−33	15	0.005828152	3.1440835	L	Caudate Tail	/

MNI, montreal neurological institute; ALE, activation likelihood estimation; BA, brodmann area; R, right; L left.

#### 3.2.3 Contrast between immediate and long-term brain activation

Due to the reported contrasts for the immediate and long-term decreased brain activation after acupuncture were less than 15 (10 for immediate activation and 4 for long-term activation), we were unable to conduct a contrast analysis for the decreased brain activation. Consequently, our analysis focused solely on increased brain activation.

**Figure 4:**
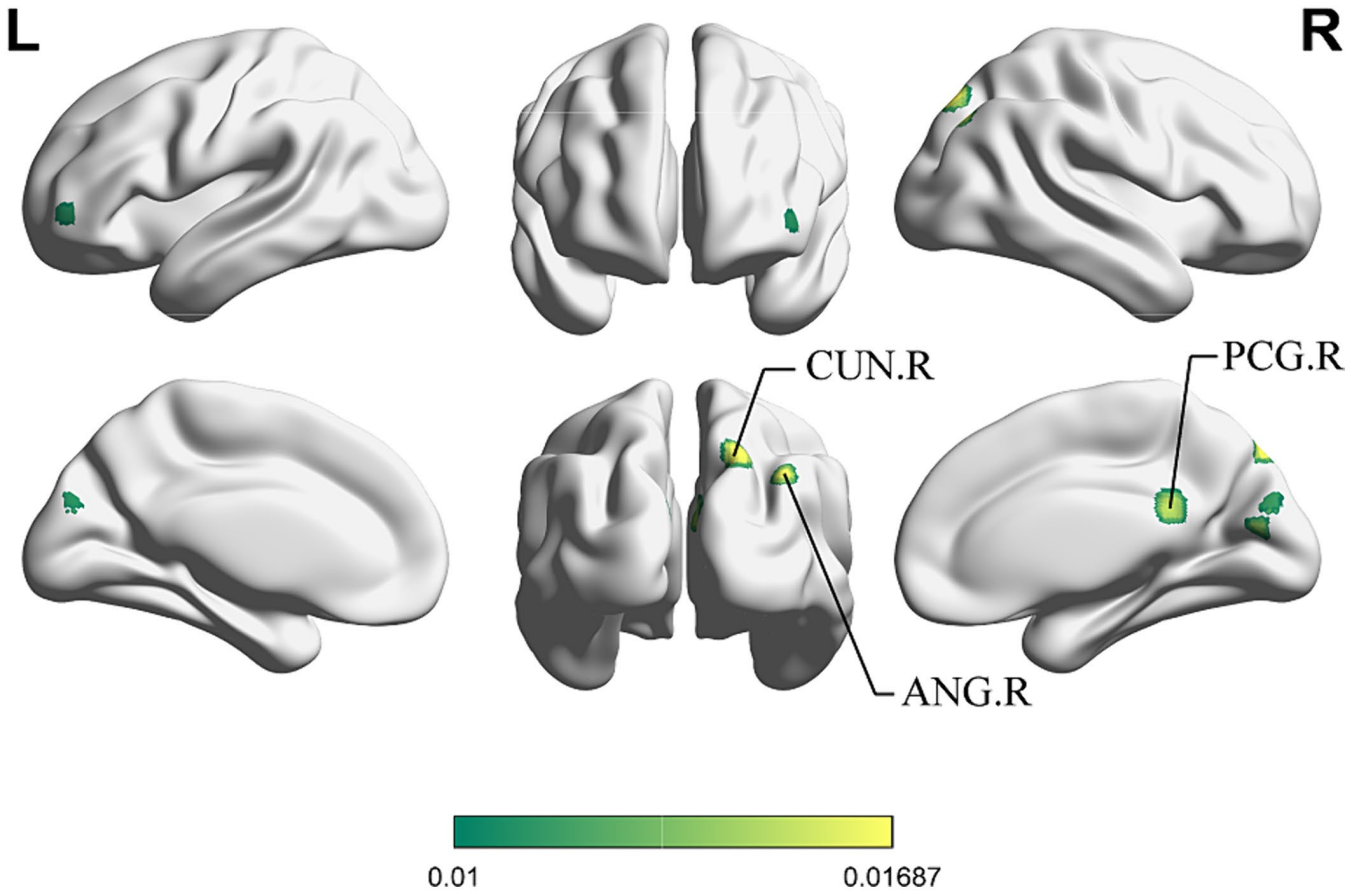
ALE map for immediate activation > long-term activation of acupuncture. The color bar represents higher ALE scores on immediate activation of acupuncture (*p* < 0.01 with 1,000 permutations). CUN, cuneus; ANG, angular gyrus; PCG, posterior cingulate gyrus; R, right; L left.

**Figure 5:**
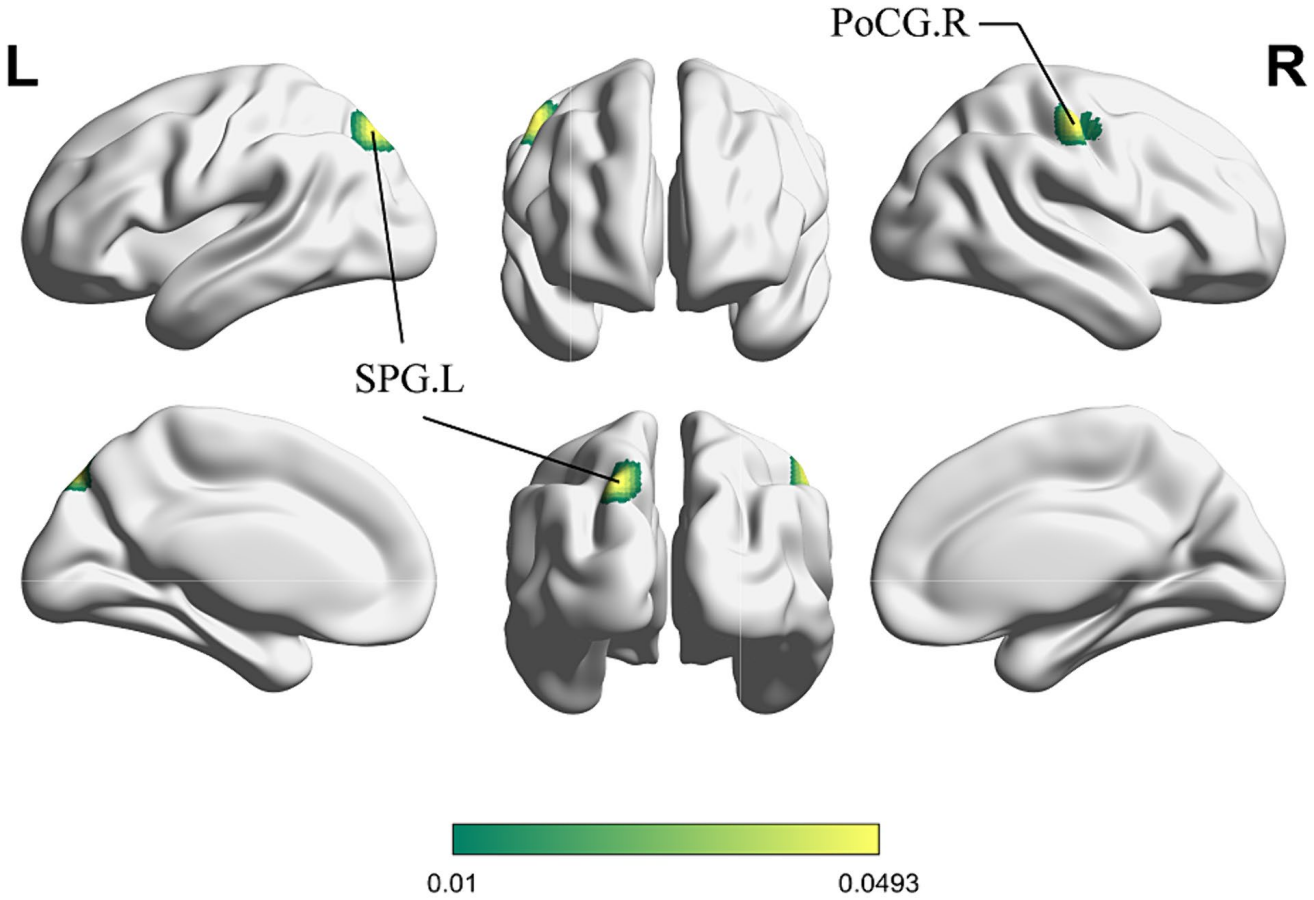
ALE map for immediate activation < long-term activation of acupuncture. The color bar represents higher ALE scores on long-term activation of acupuncture (*p* < 0.01 with 1,000 permutations). SPG, superior parietal gyrus; PoCG, postcentral gyrus; R, right; L left.

Figure 4 shows the cluster with higher ALE scores immediately after acupuncture compared to long-term acupuncture treatment in patients with IS (immediate activation > long-term activation). The cluster center is situated in the right ANG at coordinates (x, y, z = 38.2, −71, 40.8). Additionally, activations were also observed at right CUN and posterior cingulate gyrus (PCG), but without statistical significance. In Figure 5, the cluster with higher ALE scores after acupuncture treatment for one or several courses compared to immediate acupuncture treatment in patients with IS (immediate activation < long-term activation) are presented. The cluster center is located in the left superior parietal gyrus (SPG) at coordinates (x, y, z = −21.3, 76.6, 28.7). At the same time, the right postcentral gyrus (PoCG) also exhibited activation, but it was not statistically significant. Specific information about the clusters is provided in Table 5.

**Table 5:** Contrast and conjunction clusters between immediate and long-term activation after acupuncture.

Cluster	Cluster size (mm3)	Peak MNI coordinates			ALE	Z	Side	Anatomical regions	BA
		x	y	z					
Immediate effects > Long-term effects									
1	528	38.2	−71	40.8	/	2.5758293	R	Angular gyrus	19
Immediate effects < Long-term effects									
1	8,976	−21.3	−76.6	28.7	/	3.2905266	L	Superior Parietal Gyrus	7
Immediate effects ∩ Long-term effects									
1	928	−18	−88	34	0.004424779	34	L	Cuneus	18
		−18	−84	36	0.004214694	36	L	Cuneus	18
		−12	−82	40	0.002421274	40	L	Cuneus	19
2	120	−6	−76	22	0.001745806	22	L	Cuneus	18
		−4	−72	20	0.00109803	20	L	Cuneus	18
3	8	−8	−80	24	0.000912	24	L	Cuneus	18
4	8	−8	−78	26	0.001098031	26	L	Cuneus	18

MNI, montreal neurological institute; ALE, activation likelihood estimation; BA, brodmann area; R, right; L left.

#### 3.2.4 Conjunction between immediate and long-term brain activation

**Figure 6:**
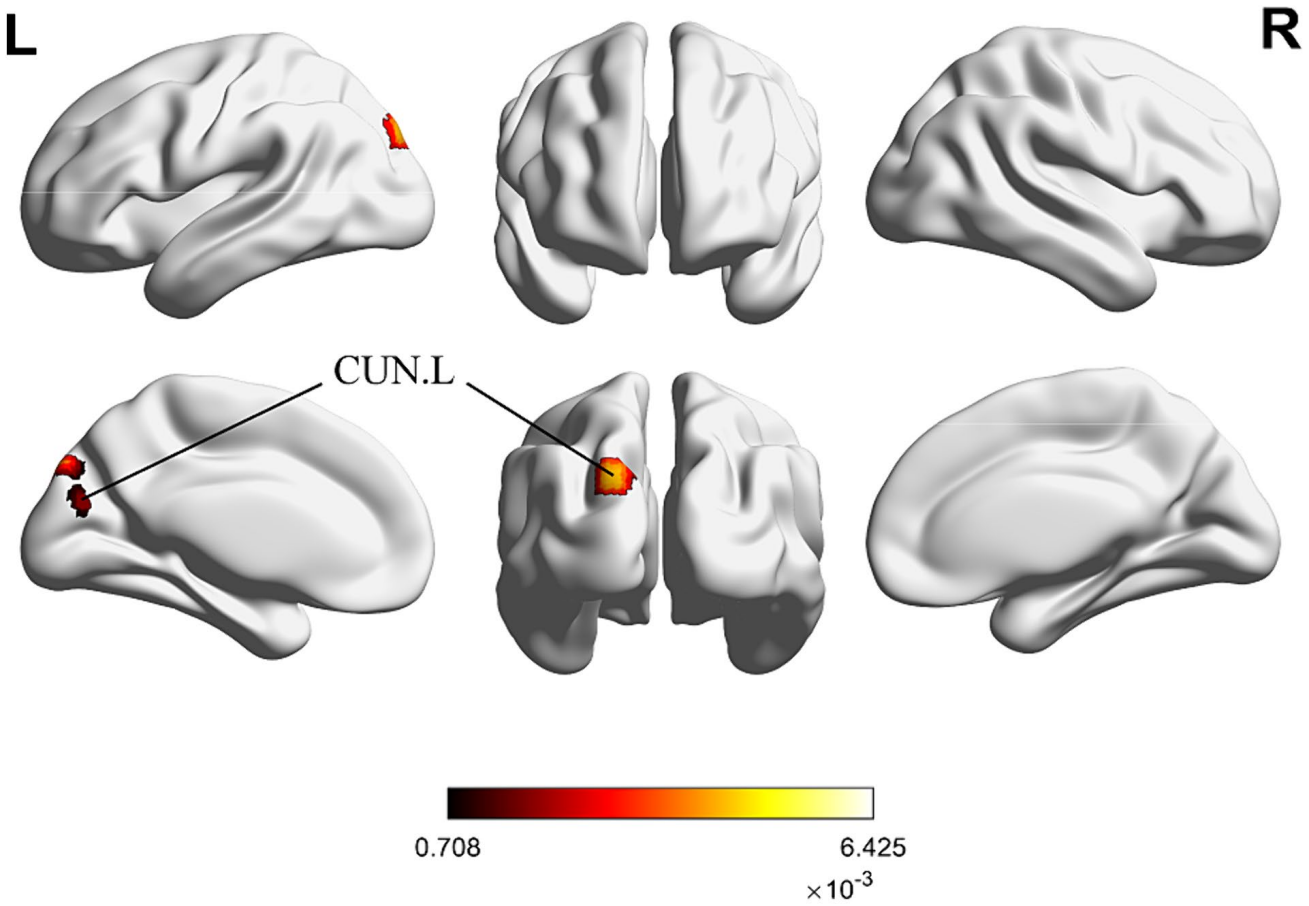
ALE map for immediate activation ∩ long-term activation of acupuncture. The color bar represents ALE scores that are jointly significant for both immediate and long-term activation of acupuncture (*p* < 0.01 with 1,000 permutations). Abbreviations: CUN, cuneus; R, right; L left.

For the same reasons outlined in 3.2.3, we conducted the conjunct analysis exclusively on the enhanced brain activation of immediate and long-term acupuncture treatment. Figure 6 depicts four clusters that are activated after both immediate and long-term acupuncture treatments (immediate activation ∩ long-term activation). The centers of four clusters are all located in the left CUN, with the largest one at coordinates (x, y, z = −18, −88, 34). Specific information on the clusters is shown in Table 5.

## 4 Discussion

As an alternative and complementary approach recommended by the World Health Organization (WHO) for stroke treatment, the efficacy mechanism of acupuncture in treating IS still requires further exploration. fMRI, with its high temporal and spatial resolution (Menon and Goodyear, 2001), is the most commonly used neuroimaging method to explore the neural mechanism of acupuncture and thus was chosen as the technique for conducting ALE meta-analysis of acupuncture brain activation in this study. In this research, we strictly adhered to inclusion and exclusion criteria, incorporating a total of 21 articles on acupuncture treatment for IS, and extracted the coordinates reported in the fMRI experiments, forming two datasets. Single dataset analysis was used to identify the immediate and long-term brain activation of acupuncture treatment for IS patients, while contrast and conjunction analysis were then used to explore the distinctions and connections between them.

Based on our findings, there appeared to be a correlation between the enhanced brain activation after immediate and long-term acupuncture in IS patients, as well as reduced brain activation. Therefore, in the following discussion, we separated the enhanced and reduced brain activation from the single dataset analysis, which may facilitate a more thorough exploration of the similarities and differences between the immediate and long-term brain activation of acupuncture.

It is noteworthy that among the 9 long-term brain activation studies on acupuncture included in the analysis, all but 1 study (Xie et al., 2013), which focused solely on brain activation, further examined patients’ recovery before and after treatment based on brain activation assessments. These studies utilized various scales depending on their different research objectives, with NIHSS, FMA, and MBI being the most commonly used. Specifically, 6 studies compared changes in patients’ scale before and after acupuncture treatment, consistently showing significant improvements in scores due to acupuncture. Additionally, 4 studies compared the effects of acupuncture treatment with traditional medication. Although (Liu H. et al., 2021) found no statistical difference in sFMA scores between acupuncture and traditional medication, other studies indicated that the improvement effects of acupuncture were more significant. Regarding treatment duration, 6 studies had a treatment period of 4 weeks, 1 had a period of 2 weeks, and 2 had a period of 1 week, with the times of acupuncture ranging from 12 to 28. Therefore, based on the clinical efficacy data, the results presented here hold particular relevance for exploring the patterns of brain functional reorganization within 1 month of acupuncture treatment. While analyzing the 12 studies on immediate brain activation by acupuncture, we noted that all these studies focused on assessing brain activity. Therefore, although these studies provided information on how acupuncture quickly affects brain activity, they did not directly reflect clinical efficacy.

### 4.1 Immediate and long-term enhanced brain activation

In the exploration of the immediate brain activation after acupuncture, we found that acupuncture can enhance the BOLD signal in various temporal, parietal, and occipital lobe regions, including the PCUN, CUN, MTG, STG, and others on both hemispheres of the brain, among which PCUN and CUN showed the most significant changes. Correspondingly, acupuncture also showed long-term enhancing activation in the brain regions centered around PCUN and CUN. As one of the most dynamically engaged regions in the cerebral cortex, PCUN and CUN are integral in the regulated brain areas crucially affected by acupuncture on the human body (Cai et al., 2018). Structurally, the PCUN is extensively connected to various brain regions in both cortex and subcortex, including the somatosensory motor cortex, visual cortex, and frontal lobe (Jitsuishi and Yamaguchi, 2023). These connections involve the PCUN in various neuroactivities, such as auditory, visual, and sensorimotor processes (Al-Ramadhani et al., 2021). The results of this study suggested that acupuncture may potentially reshape the structure and function of multiple brain regions in IS patients through the involvement of the PCUN. Simultaneously, as an important node in the Default Mode Network (DMN), the PCUN is closely associated with advanced cognitive activities like episodic memory, self-related processing, and consciousness (Raichle, 2015; Yamaguchi and Jitsuishi, 2023). It has been proven that the destruction of DMN is related to cognitive impairments after IS (Zhao et al., 2018; Wu et al., 2021), and acupuncture might facilitate improvements in cognitive function in post-IS individuals. The CUN is located on the upper surface of the occipital lobe, as an important node, the CUN participates in the activities of the visual network (VN), which primarily responsible for visual attention (Grill-Spector and Malach, 2004; Chauhan et al., 2021). The activation of CUN might suggest that acupuncture was involved in the regulation of visual processing and spatial perception after IS. Additionally, research indicated that CUN serves as a central hub for many long-distance associative fibers. Following the impact on visual function, CUN can facilitate cross-modal non-visual functions (Palejwala et al., 2021). Visual–spatial attention disorders are common symptoms of IS (Corbetta et al., 2005; Esposito et al., 2021), and acupuncture might potentially promote extensive interaction between visual and non-visual cortical areas through CUN. PCUN and CUN appeared to play crucial roles in stroke recovery, and their activation levels showed significant correlations with certain clinical scales, such as NIHSS and BI (Guo et al., 2023; Zhu et al., 2023). Studies have shown that in patients with IS, the functional connectivity of the PCUN within DMN decreases and recovers 1 month after onset, whereas the functional connectivity of the CUN within VN decreases and recovers 3 months after onset. Since the acupuncture treatment periods included in our study were within 1 month, acupuncture may potentially accelerate the functional reorganization of the DMN and VN following IS, which warrants further exploration (Zhu et al., 2023).

It’s noteworthy that in this study, immediate acupuncture induced broader activation of the brain compared to long-term activation, potentially enhancing interhemispheric communication and recovery. Kinsbourne (1977) mentioned in his hemisphere competition hypothesis that the two hemispheres of a normal brain inhibit each other through the corpus callosum. After an injury like IS, this balance is disrupted, leading not only to abnormal activity in the damaged hemisphere but also to excessive “excitement” in the healthy hemisphere due to the loss of inhibition (Carson, 2020). Our findings indicated that acupuncture may immediately facilitate communication between the two hemispheres of the brain in IS patients, and further promote the restoration of balance between the hemispheres and the recovery of function in the affected brain areas.

In contrast, the brain regions activated after long-term acupuncture were more confined, predominantly concentrated in the left hemisphere. However, these activated regions exhibited higher ALE values, which meant that IS patients had a more robust statistical result after long-term acupuncture, that is, a higher co-activation probability of the PCUN and CUN in the left hemisphere was found in previous fMRI studies. This observation suggested that long-term acupuncture may promote a relatively stable pattern of activation in the functional reorganization of the brain in IS patients. Previous studies have indicated that the patterns of brain functional reorganization in IS patients include contralateral transfer, ipsilateral functional compensation, and activation of latent pathways (Qin et al., 2022). However, most of the articles included in this study did not specify the hemisphere in which the infarct was located. Given that all articles stipulated the inclusion of patients with right-handed dominance, we reckoned that the impact of long-term acupuncture on the lateralization of brain function deserves further attention. Typically, the brain surface areas and functions of right-handers exhibit a leftward asymmetry, which is associated with functional specialization in specific cognitive processes, and possibly reflect differences in interhemispheric neural circuits (Duboc et al., 2015). In addition to the well-known asymmetry in brain regions involved in language functions (Geschwind and Levitsky, 1968), researchers have also found structural asymmetry in the frontal and occipital lobes. Specifically, the right frontal lobe extends further anteriorly than the left, while the left occipital lobe extends further posteriorly than the right (LeMay, 1976; Toga and Thompson, 2003). However, the functional relevance of this asymmetry is still unclear, and whether long-term acupuncture interventions affect the lateralization of PCUN and CUN in IS patients in relation to this structural and functional asymmetry awaits further exploration.

### 4.2 Immediate and long-term reduced brain activation

In addition to the aforementioned activation regions, we also found that acupuncture can trigger deactivation in specific brain areas. The inhibition of certain regions during brain physiological activity is crucial for maintaining overall brain function (Koush et al., 2021). Previous studies have shown that the decline in function after IS often manifests as heightened activity in corresponding brain areas (Dickerson et al., 2004; Paradiso et al., 2013). The deactivation in specific brain regions by acupuncture may be associated with a reduced reliance on compensation in these areas, and may also involve the restoration of overall brain function.

Our results indicated that both immediate and long-term brain deactivation after acupuncture in IS patients are associated with emotion and cognition. A reduced BOLD signal cluster formed in the MFG immediately after acupuncture, while the deactivation occurred in the INS and HIP after a period of acupuncture treatment. From a broader perspective, the brain region of decreased activity immediately after acupuncture is located in the dorsolateral prefrontal cortex (dlPFC), while those after long-term treatment are in the limbic system. The dlPFC is considered a core component of human identity, playing a crucial role in high-level cognitive abilities (Barbas, 2015; Ma et al., 2022). Similarly, the limbic system is essential for emotional responses, cognition, and memory (Torrico and Abdijadid, 2024). For stroke patients, increased brain activity in these regions often accompanies a decrease in emotional experience (Paradiso et al., 2013). Abnormal activation of dlPFC has been observed following a IS, especially in post-stroke depression, where the lesion location maps to a depression circuit centered on the left dlPFC (Stewart et al., 2016; Padmanabhan et al., 2019). Additionally, the limbic system, represented by the HIP, experienced continuous secondary degeneration of neurons following an IS, triggering a series of symptoms, particularly associated with the onset of cognitive impairment, irrespective of the lesion’s location (Haque et al., 2019). Acupuncture may reverse these trends. It’s worth noting that although both dlPFC and the limbic system can regulate emotion and cognition, their regulatory patterns differ. The limbic system is primarily associated with the generation of emotions and memories, whereas the prefrontal lobe is more inclined to play a key role in regulating emotions and cognition (Kebets et al., 2021). Integrating the findings of this study, the immediate and long-term brain deactivation after acupuncture may involve different regulatory mechanisms for the emotion and cognition of IS patients, which require further exploration.

### 4.3 Contrast and conjunction between immediate and long-term brain activation

Based on the above results, a further contrast and conjunct analysis was conducted on the activated brain regions in IS patients between immediate and long-term acupuncture. It was observed that compared to long-term acupuncture, there was a more significant activation around the right ANG immediately after acupuncture. ANG is located at the junction of the temporal, parietal, and occipital lobes. Due to its strategic location and rich connectivity, various sensory (auditory, visual, and somatosensory) information is combined and integrated here (Seghier, 2013). For a long time, ANG has also been considered a key node in the DMN (Raichle, 2015; Vatansever et al., 2017), associated with various cognitive functions (Schurz et al., 2017; Bonnici et al., 2018; Lewis et al., 2019; Humphreys et al., 2021). Numerous neuroimaging studies have recorded abnormal structures and functions of ANG in various neurological and psychiatric disorders (Wang X. et al., 2018; Zeng et al., 2021; Pico-Perez et al., 2022). However, recent research indicated that ANG is a cross-modal hub involved in the activity of multiple functional networks (Song et al., 2023). In contrast to the relatively limited activated brain regions with long-term acupuncture, ANG may become an important link in promoting the widespread activation of bilateral temporal, parietal and occipital regions immediately after acupuncture through cross-network information exchange and integration. Additionally, higher ALE values were observed in the right PCG immediately after acupuncture compared to long-term acupuncture. Although there is no statistical significance, the PCG, as a brain region responsible for receiving spatial and action-related information from the parietal cortex (Rolls, 2019), plays a core role in supporting internal orientation cognition and is also a key node of the DMN (Leech and Sharp, 2014), further highlighting that the immediate brain activation of acupuncture for IS involve a broader range of modulation and improvement in cognitive-related neural activities compared to the long-term activation.

Correspondingly, in the SPG, we observed an activated cluster where long-term acupuncture resulted in higher ALE scores compared to immediately after acupuncture, as well as an activation trend in PoCG, but without statistical significance. According to statistics, more than half of the patients experienced impaired sensory function after an IS, possibly linked to functional damage or reduction in parietal lobe-related brain areas (Sullivan and Hedman, 2008; Chen et al., 2023). The parietal cortex acts as a hub for sensory-motor integration, particularly the superior parietal region, with intricate fiber connections (Van de Winckel et al., 2005; Nakashita et al., 2008; Sereno and Huang, 2014). Both the SPG and PoCG are responsible for somatic sensation. The SPG interconnects simple neural impulses from the PoCG for further analysis and integration, and is involved in the sensation related to intricate activities of the contralateral limbs (Berlucchi and Vallar, 2018). Lesions affecting the SPG and PoCG after an IS can lead to complex sensory disturbances in the corresponding areas of the contralateral limbs (Kumral et al., 2023). The results of this study suggest that, compared to immediately after acupuncture, long-term acupuncture may have a promoting effect on the recovery of self-sensation in IS patients, especially in complex sensations. This reaffirms previous research findings (Li et al., 2021).

In the subsequent conjunction analysis, we identified a statistically significant intersection in the left CUN among the brain regions activated in IS patients after both immediate and long-term acupuncture. The left CUN may serve as a key brain region where immediate acupuncture modulation accumulates and transfers to a prolonged change. This finding is introduced for the first time in this study, further validating the earlier mention of acupuncture’s potential to modulate visual processing and spatial perception. The recovery of CUN functional activity might also be important in the rehabilitation treatment of IS (Jiang et al., 2019). Studies have shown that IS patients increasingly rely on visual feedback during rehabilitation, and functional topological changes in the CUN and surrounding occipital cortex might serve as prognostic biomarkers for subacute IS (Huang et al., 2022). Whether acupuncture can also facilitate this process and contribute to functional recovery in IS patients requires further evidence.

Due to the insufficient number of reported contrasts included, we did not conduct a contrast and conjunct analysis on the immediate and long-term brain deactivation after acupuncture. However, based on the results of the single dataset analysis, both immediate and long-term acupuncture exhibit independent deactivated brain regions, with no overlapping areas. By analyzing these results, we can preliminarily glean the similarities and differences in the immediate and long-term activation patterns of acupuncture on reducing brain activity in IS. However, these analytical findings still require further support from additional data.

### 4.4 Limitations

This study has several limitations that need to be addressed. Firstly, the ALE meta-analysis is a coordinate-based method, which means that the results we obtained were the co-activation probabilities of coordinates in different neuroimaging studies. These results neither incorporated inter-group or condition differences among the experiments, nor did they reveal variations in the degree of activation/deactivation in specific brain areas (Muller et al., 2018). Secondly, as FC analysis is a prevalent method in current acupuncture neuroimaging research, and given that a significant portion of the participants in this study had sensory-motor impairments, we chose to include whole-brain voxel-level FC studies with the primary sensory-motor cortex of the affected side as the region of interest (ROI). This was done to minimize bias caused by excluding numerous studies that used different imaging data analysis methods, but it might lead to inflated significance in that specific region in the results (Muller et al., 2018). Thirdly, post-acupuncture states after one or more sessions tend to incline more towards a resting state, while immediately after acupuncture, the state leans more towards a task state. Although we specified that patients did not receive any instructions and maintained a resting state during acupuncture to minimize the external environment’s impact on the brain, there might still be differences in the baseline brain activity between the two conditions. Fourthly, patients’ brains undergo time-dependent plasticity after IS (Zhu et al., 2023). This study did not compare the brain recovery of IS patients who had not undergone acupuncture treatment, and the long-term brain activation in the results might include the inherent recovery of the patients themselves. Fifth, although some studies have shown that with sample sizes of 12 to 20 in fMRI research, 80% of brain activity clusters are reproducible (Desmond and Glover, 2002; Murphy and Garavan, 2004), the sample sizes in some of the literature included in this study are still small, which may impact the generalizability of our findings and conclusions. Finally, according to traditional meridian theory, the therapeutic effects of acupoints vary. The literature included in this study generally employed different combinations of acupoints based on the specific symptoms and stages of IS in patients, which brought heterogeneity in intervention and lesions, thus, the final results can only represent the general activation patterns of acupuncture for IS.

## 5 Conclusion

Through an ALE meta-analysis, we systematically analyzed previous fMRI studies on acupuncture for IS. As expected, whether immediately after acupuncture or following long-term acupuncture treatment, there existed a relatively stable pattern of altered brain activation. Our findings preliminarily unveiled the intricate relationship between the immediate and long-term activation of acupuncture. Both immediate and long-term activation patterns exhibited enhancement effects in brain regions centered around PCUN and CUN, which possibly served as the foundation for the cross-regional and multi-network synergistic regulation in IS patients, and further participated in regulating various advanced cognitive and perceptual functions, including memory, attention, visual processing, and spatial perception. Particularly, the left CUN may serve as a key brain region where immediate acupuncture modulation accumulates and transfers to a prolonged change. Additionally, compared to immediately after acupuncture, long-term acupuncture resulted in more localized activation areas but with higher co-activation probability. The distinct deactivation areas suggested that the two patterns may involve different regulatory mechanisms on emotions and cognition in IS patients.

The regulation of brain function by acupuncture after IS is complex. The immediate effects of acupuncture may be closely related to neural signal transmission, whereas the long-term effects may additionally involve signal transmission through hormones and cytokines (Cui et al., 2021). Our study results demonstrate, to some extent, the immediate and long-term brain activation of acupuncture in IS patients on a macro scale. The effects of acupuncture on brain function and structure, from the macro to the meso and micro scales, as well as the interaction mechanisms of cells and cytokines within the neuro-endocrine-immune system, still warrant further exploration. Such multi-scale and multi-system studies will enable a more comprehensive understanding of the mechanisms underlying acupuncture treatment for IS and provide more detailed scientific evidence for its clinical application.

## Data availability statement

The original contributions presented in the study are included in the article/Supplementary material, further inquiries can be directed to the corresponding authors.

## Author contributions

YZ: Conceptualization, Data curation, Formal analysis, Investigation, Methodology, Software, Writing – original draft, Writing – review & editing. HL: Data curation, Supervision, Writing – review & editing. XR: Data curation, Resources, Writing – review & editing. JZ: Software, Visualization, Writing – review & editing. YW: Software, Visualization, Writing – review & editing. CZ: Conceptualization, Funding acquisition, Validation, Writing – review & editing. XZ: Conceptualization, Supervision, Validation, Writing – review & editing.

## Footnotes

^1^ https://pubmed.ncbi.nlm.nih.gov/

^2^ https://www.cochranelibrary.com/

^3^ https://www.embase.com/

^4^ https://www.webofscience.com/

^5^ https://www.cnki.net/

^6^ http://www.sinomed.ac.cn/

^7^ http://qikan.cqvip.com/

^8^ http://wanfangdata.com.cn/

^9^ https://training.cochrane.org/handbook

^10^ http://brainmap.org/

^11^ http://www.nitrc.org/projects/bnv/
