# Supplementary material for: Immediate and long-term brain activation of acupuncture on ischemic stroke patients: an ALE meta-analysis of fMRI studies
Source: Front Neurosci. 2024 Jul 19;18:1392002. doi: 10.3389/fnins.2024.1392002 (PMC11294246; doi:10.3389/fnins.2024.1392002)
Supplement: Supplementary file 1 [file Data_Sheet_1.docx]

Supplementary Material

## Supplementary Table 1. A PRISMA-A checklist

| **Subjects** | **PRISMA for Acupuncture** | | **Reported on page #** |
| --- | --- | --- | --- |
| ***Title*** |  |  |  |
| **Title** | 1^*^ Identify the report as a systematic review, meta-analysis, or both; if applicable, state the specific type of acupuncture treatment, such as manual acupuncture or electroacupuncture. | | 1 |
| ***Abstract*** |  |  |  |
| **Structured summary** | 2^†^ Provide a structured summary including, as applicable: background; objectives; data sources; study eligibility criteria, participants, and interventions; study appraisal and synthesis methods; results limitations; conclusions and implications of key findings; systematic review registration number. | | 1, 2 |
| ***Introduction*** |  |  |  |
| **Rationale** | 3^*^ Describe the rationale for what is already known about acupuncture for the target condition in the background; if applicable, state what is already known about the specific types of acupuncture to be studied, and describe whether there is any difference of the effects among different types of acupuncture. | | 2, 3 |
| **Objectives** | 4^†^ Provide an explicit statement of questions being addressed with reference to participants, interventions, comparisons, outcomes, and study design (PICOS) | | 3 |
| ***Methods*** |  |  |  |
| **Protocol and registration** | 5^†^ Indicate if a review protocol exists, if and where it can be accessed (e.g., web address), and, if available, provide registration information including registration number. | | 4 |
| **Eligibility criteria** | 6^†^ Specify study characteristics (e.g., PICOS, length of follow-up) and report characteristics (e.g., years considered, language, publication status) used as criteria for eligibility, giving rationale.  6a.1^‡^ Describe the diagnostic criteria of the target condition in Western medicine.  6a.2^‡^ If applicable, describe the diagnostic criteria in terms of Traditional Medicine, such as Traditional Chinese Medicine.  6b^‡^ Describe the types of acupuncture to be included, such as traditional acupuncture, electroacupuncture, or fire acupuncture.  6c^‡^ If applicable, report measures for therapeutic effects using the terminology of either traditional medicine (e.g. syndrome score for syndrome remission) or Western medicine (e.g. pain intensity). | | 4 |
|  |  |  |  |
|  |  |  |  |
|  |  |  |  |
| **Information sources** | 7^*^ Describe all sources of information (e.g., databases with dates of coverage, contact with study authors to identify additional studies) in the search, and report the date of the last search. If applicable, report the databases or complementary search methods for acupuncture or traditional medicine. | | 4 |
| **Search** | 8^*^ Present full electronic search strategy for at least one commonly used database (e.g. MEDLINE), including any limits used, such that it could be repeated. If applicable, include the full search strategy for at least a Western and a traditional medicine database for each systematic review where both were used. | | Supplementary Material |
| **Study selection** | 9^†^ State the process for selecting studies (i.e., screening, eligibility, included in systematic review, and, if applicable, included in the meta-analysis). | | 4 |
| **Data collection**  **process** | 10^†^ Describe method of data extraction from reports (e.g., piloted forms, independently, in duplicate) and any processes for obtaining and confirming data from investigators. | | 4 |
| **Data items** | 11^*^ List and define all variables for which data were sought (e.g., PICOS, funding sources) and any assumptions and simplifications made; describe data items about details of acupuncture interventions and controls (**e.g.,** sham acupuncture) referring to TIDieR when applicable. | | 4, 5 |
| **Risk of bias in**  **individual studies** | 12^†^ Describe methods used for assessing risk of bias of individual studies (including specification of whether this was done at the study or outcome level), and how this information is to be used in any data synthesis. | | 4, 5 |
| **Summary measures** | 13^†^ State the principal summary measures (e.g., risk ratio, difference in means). | | 5 |
| **Synthesis of results** | 14^†^ Describe the methods of handling data and combining results of studies, if done, including measures of consistency (e.g., I^2^) for each meta-analysis. | | 5 |
| **Risk of bias across**  **studies** | 15^†^ Specify any assessment of risk of bias that may affect the cumulative evidence (e.g., publication bias, selective reporting within studies). | | 5 |
| **Additional analyses** | 16^†^ Describe methods of additional analyses (e.g., sensitivity or subgroup analyses, meta-regression), if done, indicating which were pre-specified. | | NA |
| ***Results*** |  |  |  |
| **Study selection** | 17^†^ Give numbers of studies screened, assessed for eligibility, and included in the review, with reasons for exclusions at each stage, ideally with a flow diagram. | | 6 |
| **Study characteristics** | 18* For each study, present characteristics that were extracted (e.g., study size, PICOS, follow-up period) and provide the citations of the included studies. Summarize details of the acupuncture intervention for each study in a table referring to TIDieR**.**  18a^‡^ Describe details of “De-qi” after acupuncture reported in the included studies. | | 6 |
| **Risk of bias within**  **studies** | 19^†^ Present data on risk of bias of each study and, if available, any outcome-level assessment(see item 12). | | 6 |
| **Results of individual**  **studies** | 20^†^ For all outcomes considered (benefits or harms), present, for each study: (a) simple summary data for each intervention group and (b) effect estimates and confidence intervals, ideally with a forest plot. | | 6, 7 |
| **Synthesis of results** | 21^†^ Present results of each meta-analysis done, including confidence intervals and measures of consistency. | | 6, 7 |
| **Risk of bias across**  **studies** | 22^†^ Present results of any assessment of risk of bias across studies (see item 15). | | 6 |
| **Additional analysis** | 23^†^ Give results of additional analyses, if done (e.g., sensitivity or subgroup analyses, meta-regression [see item 16]). | | NA |
| ***Discussion*** |  |  |  |
| **Summary of evidence** | 24^†^ Summarize the main findings including the strength of evidence for each main outcome; consider their relevance to key groups (e.g., health care providers, users, and policy  makers). | | 5-13 |
| **Limitations** | 25^†^ Discuss limitations at study and outcome level (e.g., risk of bias), and at review level (e.g., incomplete retrieval of identified research, reporting bias). | | 13 |
| **Conclusions** | 26^†^ Provide a general interpretation of the results in the context of other evidence, and implications for future research. | | 13 |
| ***Funding*** |  |  |  |
| **Funding** | 27^†^ Describe sources of funding for the systematic review and other support (e.g., supply of data); role of funders for the systematic review. | | 14 |

Note: * modified original item ^†^ unmodified item from PRISMA ^‡^ new extended item

## Supplementary Table 2. Search terms and results in different databases

| Database | Search Terms | Search Field | Search Results |
| --- | --- | --- | --- |
| PubMed | ((("Ischemic Stroke"[Mesh]) OR ((((((((("Ischemic Stroke") OR ("Cerebral Infarction")) OR ("CVA")) OR ("Cerebrovascular Accident")) OR ("Cerebrovascular Attack")) OR ("Cerebral Embolism")) OR ("Cerebrovascular Apoplexy")) OR ("Apoplexy")) OR ("Brain Vascular Accident"))) AND (("Magnetic Resonance Imaging"[Mesh]) OR (((((((("Magnetic Resonance Imaging") OR ("MRI")) OR ("fMRI")) OR ("Functional Magnetic Resonance Imaging")) OR ("Functional MRI")) OR ("BOLD")) OR ("Blood Oxygenation Level Dependent")) OR ("Neuroimaging")))) AND (("Acupuncture"[Mesh]) OR (((((("Acupuncture") OR ("Electroacupuncture")) OR ("Acupuncture Therapy")) OR ("Auriculotherapy")) OR ("Scalp Acupuncture")) OR ("Acupuncture Points"))) | All Fields | 109 |
| Cochrane | #1. MeSH descriptor: [Ischemic Stroke] explode all trees  #2. (Ischemic Stroke):ti,ab,kw OR (Cerebral Infarction):ti,ab,kw OR (CVA):ti,ab,kw OR (Cerebrovascular Accident):ti,ab,kw OR (Cerebrovascular Attack):ti,ab,kw  #3. (Cerebral Embolism):ti,ab,kw OR (Cerebrovascular Apoplexy):ti,ab,kw OR (Apoplexy):ti,ab,kw OR (Brain Vascular Accident):ti,ab,kw  #4. #1 OR #2 OR #3  #5. MeSH descriptor: [Magnetic Resonance Imaging] explode all trees  #6. (Magnetic Resonance Imaging):ti,ab,kw OR (MRI):ti,ab,kw OR (fMRI):ti,ab,kw OR (Functional Magnetic Resonance Imaging):ti,ab,kw OR (Functional MRI):ti,ab,kw  #7. (BOLD):ti,ab,kw OR (Blood Oxygenation Level Dependent):ti,ab,kw OR (Neuroimaging):ti,ab,kw  #8. #5 OR #6 OR #7  #9. MeSH descriptor: [Acupuncture] explode all trees  #10. (Acupuncture):ti,ab,kw OR (Electroacupuncture):ti,ab,kw OR (Acupuncture Therapy):ti,ab,kw OR (Auriculotherapy):ti,ab,kw OR (Scalp Acupuncture):ti,ab,kw  #11 (Acupuncture Points):ti,ab,kw  #12. #9 OR #10 OR #11  #13. #4 AND #8 AND #12 | Title, Abstract, Keywords | 77 |
| WOS | #4. #3 AND #2 AND #1  #3. Acupuncture (All Fields) or Electroacupuncture (All Fields) or Acupuncture Therapy (All Fields) or Auriculotherapy (All Fields) or Scalp Acupuncture (All Fields) or Acupuncture Points (All Fields)  #2. MRI (All Fields) or fMRI (All Fields) or Magnetic Resonance Imaging (All Fields) or Functional Magnetic Resonance Imaging (All Fields) or Functional MRI (All Fields) or BOLD (All Fields) or Blood Oxygenation Level Dependent (All Fields) or Neuroimaging (All Fields)  #1. Ischemic Stroke (All Fields) or Cerebral Infarction (All Fields) or CVA (All Fields) or Cerebrovascular Accident (All Fields) or Cerebrovascular Attack (All Fields) or Cerebral Embolism (All Fields) or Cerebrovascular Apoplexy (All Fields) or Apoplexy (All Fields) or Brain Vascular Accident (All Fields) | All Fields | 167 |
| EMBASE | #10. #3 AND #6 AND #9  #9. #7 OR #8  #8. 'acupuncture':ti,ab,kw OR 'electroacupuncture':ti,ab,kw OR 'acupuncture therapy':ti,ab,kw OR 'auricular acupuncture':ti,ab,kw OR 'scalp acupuncture':ti,ab,kw OR 'acupuncture point':ti,ab,kw  #7. 'acupuncture'/exp  #6. #4 OR #5  #5. 'functional magnetic resonance imaging':ti,ab,kw OR 'mri':ti,ab,kw OR 'fmri':ti,ab,kw OR 'magnetic resonance imaging':ti,ab,kw OR 'functional mri':ti,ab,kw OR 'bold':ti,ab,kw OR 'blood oxygenation level dependent':ti,ab,kw OR 'neuroimaging':ti,ab,kw  #4. 'functional magnetic resonance imaging'/exp  #3. #1 OR #2  #2. 'cerebrovascular accident':ti,ab,kw OR 'ischemic stroke':ti,ab,kw OR 'cerebral infarction':ti,ab,kw OR cva:ti,ab,kw OR 'cerebrovascular attack':ti,ab,kw OR 'cerebral embolism':ti,ab,kw OR 'cerebrovascular apoplexy':ti,ab,kw OR 'apoplexy':ti,ab,kw OR 'brain vascular accident':ti,ab,kw  #1. 'cerebrovascular accident'/exp | Title, Abstract, Keywords | 159 |
| CNKI | TKA=('中风'+'脑梗死'+'脑梗塞'+'卒中'+'缺血性脑卒中'+'脑血管意外') AND TKA=('MRI'+'fMRI'+'磁共振'+'功能磁共振'+'BOLD'+'多模式MRI'+'神经影像学') AND TKA=('针灸'+'针刺'+'电针'+'针灸疗法'+'耳针'+'头针'+'穴位') | Title, Abstract, Keywords | 347 |
| VIP | (((((((任意字段=中风 OR 任意字段=脑梗死) OR 任意字段=脑梗塞) OR 任意字段=卒中) OR 任意字段=缺血性脑卒中) OR 任意字段=脑血管意外) AND ((((((任意字段=MRI OR 任意字段=fMRI) OR 任意字段=磁共振) OR 任意字段=功能磁共振) OR 任意字段=BOLD) OR 任意字段=多模式MRI) OR 任意字段=神经影像学)) AND ((((((任意字段=针灸 OR 任意字段=针刺) OR 任意字段=电针) OR 任意字段=针灸疗法) OR 任意字段=耳针) OR 任意字段=头针) OR 任意字段=穴位)) | All Fields | 405 |
| WF | (中风 OR 脑梗死 OR 脑梗塞 OR 卒中 OR 缺血性脑卒中 OR 脑血管意外) AND (MRI OR fMRI OR 磁共振 OR 功能磁共振 OR BOLD OR 多模式MRI OR 神经影像学) AND (针灸 OR 针刺 OR 电针 OR 针灸疗法 OR 耳针 OR 头针 OR 穴位) | All Fields | 514 |
| CBM | (("中风"[不加权:扩展] OR ("中风"[常用字段:智能] OR "脑梗死"[常用字段:智能] OR "脑梗塞"[常用字段:智能] OR "卒中"[常用字段:智能] OR "缺血性脑卒中"[常用字段:智能] OR "脑血管意外"[常用字段:智能])) AND (("磁共振成像"[不加权:扩展]) OR ("磁共振成像"[常用字段:智能] OR "MRI"[常用字段:智能] OR "fMRI"[常用字段:智能] OR "功能磁共振"[常用字段:智能] OR "BOLD"[常用字段:智能] OR "多模式MRI"[常用字段:智能] OR "神经影像学"[常用字段:智能])) AND (("针灸疗法"[不加权:扩展]) OR ("针灸疗法"[常用字段:智能] OR "针刺"[常用字段:智能] OR "电针"[常用字段:智能] OR "针灸"[常用字段:智能] OR "耳针"[常用字段:智能] OR "头针"[常用字段:智能] OR "穴位"[常用字段:智能])) | All Fields | 430 |

## Supplementary Table 3. Author judgments for ROB assessment

| Study ID | Domain | Judgment |
| --- | --- | --- |
| HL Xiao et al. 2012 | Selection bias | Unclear risk "no mention of random methodology and allocation concealment" |
|  | Performance bias | Unclear risk "no mention of blinding" |
|  | Detection bias | Unclear risk "no mention of blinding" |
|  | Attrition bias | Unclear risk "number of persons not reported for inclusion in the analysis of results" |
| J Qi et al. 2014 | Selection bias | Unclear risk "no mention of random methodology and allocation concealment" |
|  | Performance bias | Unclear risk "no mention of blinding" |
|  | Detection bias | Unclear risk "no mention of blinding" |
| P Wu et al. 2017 | Performance bias | Unclear risk "no mention of blinding" |
|  | Detection bias | Unclear risk "no mention of blinding" |
| HC Liu et al. 2020 | Selection bias | Unclear risk "no mention of allocation concealment" |
|  | Performance bias | Unclear risk "no mention of blinding" |
|  | Detection bias | Unclear risk "no mention of blinding" |
| K Fan et al. 2021 | Selection bias | Unclear risk "no mention of random methodology and allocation concealment" |
|  | Performance bias | Unclear risk "no mention of blinding" |
|  | Detection bias | Unclear risk "no mention of blinding" |
| HC Liu et al. 2021 | Selection bias | Unclear risk "no mention of allocation concealment" |
|  | Performance bias | Unclear risk "no mention of blinding" |
|  | Detection bias | Unclear risk "no mention of blinding" |
| Y Liu et al. 2021 | Selection bias | Unclear risk "no mention of allocation concealment" |
| ZF Ye et al. 2018 | Selection bias | Unclear risk "no mention of allocation concealment" |
|  | Performance bias | Unclear risk "no mention of blinding" |
|  | Detection bias | Unclear risk "no mention of blinding" |
|  | Other bias | Unclear risk "incomplete information makes it difficult to determine whether there is significant bias." |
| MK Li et al. 2015 | Selection bias | High risk "Group according to deqi"& Unclear risk"no mention of allocation concealment" |
|  | Performance bias | Unclear risk "no mention of blinding" |
|  | Detection bias | Unclear risk "no mention of blinding" |

## Supplementary Table 4. Author judgments for MINORS

| Study ID | Type | Q1 | Q2 | Q3 | Q4 | Q5 | Q6 | Q7 | Q8 | Q9 | Q10 | Q11 | Q12 | Total points |
| --- | --- | --- | --- | --- | --- | --- | --- | --- | --- | --- | --- | --- | --- | --- |
| P Dong et al. 2010 | Double or multiple-arm trial | 2 | 2 | 2 | 2 | 2 | 2 | 0 | 0 | 2 | 0 | 0 | 2 | 16 |
| JQ Chen et al. 2011 | Single-arm trial | 2 | 1 | 2 | 2 | 2 | 2 | 2 | 0 | — | — | — | — | 13 |
| Y Zheng et al. 2012 | Single-arm trial | 2 | 1 | 2 | 2 | 2 | 2 | 2 | 0 | — | — | — | — | 13 |
| SY Cho et al. 2013 | Double or multiple-arm trial | 2 | 1 | 2 | 2 | 2 | 2 | 2 | 0 | 2 | 1 | 1 | 2 | 19 |
| XM Xie et al. 2013 | Double or multiple-arm trial | 2 | 2 | 2 | 2 | 2 | 2 | 2 | 0 | 2 | 1 | 2 | 2 | 21 |
| Y Huang et al. 2013 | Single-arm trial | 2 | 2 | 2 | 2 | 2 | 2 | 2 | 0 | — | — | — | — | 14 |
| W Wang et al. 2016 | Double or multiple-arm trial | 2 | 2 | 2 | 2 | 2 | 2 | 2 | 0 | 2 | 2 | 2 | 2 | 22 |
| GF Zhang et al. 2017 | Double or multiple-arm trial | 2 | 2 | 2 | 2 | 2 | 2 | 2 | 0 | 2 | 0 | 0 | 2 | 18 |
| CH Fu et al. 2019 | Double or multiple-arm trial | 2 | 2 | 2 | 2 | 2 | 2 | 2 | 0 | 2 | 2 | 2 | 2 | 22 |
| SQ Chen et al. 2020 | Single-arm trial | 2 | 2 | 2 | 2 | 2 | 2 | 2 | 0 | — | — | — | — | 14 |
| XQ Yi et al. 2021 | Double or multiple-arm trial | 2 | 2 | 2 | 2 | 2 | 2 | 2 | 0 | 2 | 1 | 2 | 2 | 21 |
| J Peng et al. 2023 | Double or multiple-arm trial | 2 | 1 | 2 | 2 | 2 | 2 | 2 | 0 | 2 | 1 | 2 | 2 | 20 |

The first subscale of 8 items related to non-comparative studies, whereas all 12 items were relevant to comparative studies. The items are scored 0 (not reported), 1 (reported but inadequate) or 2 (reported and adequate). The global ideal score being 16 for non-comparative studies and 24 for comparative studies.

## Supplementary Table 5. Author judgments for content integrity

| Study ID | Q1 | Q2 | Q3 | Q4 | Q5 | Q6 | Q7 | Q8 | Q9 | Q10 | Total points |
| --- | --- | --- | --- | --- | --- | --- | --- | --- | --- | --- | --- |
| P Dong et al. 2010 | 1 | 0.5 | 0.5 | 0.5 | 0 | 1 | 1 | 1 | 1 | 0 | 6.5 |
| JQ Chen et al. 2011 | 1 | 1 | 1 | 1 | 0 | 1 | 1 | 1 | 1 | 0 | 8 |
| Y Zheng et al. 2012 | 1 | 1 | 1 | 1 | 0 | 1 | 1 | 1 | 1 | 0 | 8 |
| HL Xiao et al. 2012 | 1 | 1 | 1 | 1 | 0 | 1 | 1 | 1 | 1 | 0 | 8 |
| SY Cho et al. 2013 | 0 | 1 | 1 | 1 | 1 | 1 | 1 | 1 | 1 | 0 | 8 |
| XM Xie et al. 2013 | 1 | 1 | 1 | 0.5 | 0 | 1 | 1 | 1 | 1 | 0 | 7.5 |
| Y Huang et al. 2013 | 0 | 1 | 1 | 1 | 0 | 1 | 1 | 1 | 1 | 0 | 7 |
| J Qi et al. 2014 | 1 | 1 | 1 | 1 | 0 | 1 | 1 | 1 | 1 | 0 | 8 |
| MK Li et al. 2015 | 1 | 1 | 1 | 1 | 0 | 1 | 1 | 1 | 1 | 0 | 8 |
| W Wang et al. 2016 | 0 | 1 | 1 | 0.5 | 1 | 1 | 1 | 1 | 1 | 0 | 7.5 |
| P Wu et al. 2017 | 1 | 1 | 1 | 1 | 1 | 1 | 1 | 1 | 1 | 1 | 10 |
| GF Zhang et al. 2017 | 1 | 1 | 1 | 1 | 1 | 1 | 1 | 1 | 1 | 0 | 9 |
| ZF Ye et al. 2018 | 1 | 1 | 1 | 0 | 1 | 1 | 1 | 0 | 1 | 0 | 7 |
| CH Fu et al. 2019 | 1 | 1 | 1 | 0 | 1 | 1 | 1 | 1 | 1 | 1 | 9 |
| SQ Chen et al. 2020 | 1 | 1 | 1 | 1 | 0 | 1 | 1 | 1 | 1 | 1 | 9 |
| HC Liu et al. 2020 | 1 | 1 | 1 | 0.5 | 0 | 1 | 1 | 1 | 1 | 0 | 8.5 |
| K Fan et al. 2021 | 1 | 1 | 1 | 1 | 1 | 1 | 1 | 1 | 1 | 0 | 9 |
| HC Liu et al. 2021 | 1 | 1 | 1 | 0.5 | 0 | 1 | 1 | 1 | 1 | 0 | 8.5 |
| Y Liu et al. 2021 | 1 | 1 | 1 | 1 | 1 | 1 | 1 | 1 | 1 | 1 | 10 |
| XQ Yi et al. 2021 | 0 | 1 | 1 | 0.5 | 1 | 1 | 1 | 1 | 1 | 1 | 8.5 |
| J Peng et al. 2023 | 0 | 1 | 2 | 0.5 | 1 | 1 | 1 | 1 | 1 | 1 | 8.5 |

0/0.5/1 score for each item; Totally 10 points. Give 0.5 points if some of the criteria are met.

**Category 1: sample characteristics**

1. Patients are evaluated with specific standardized diagnostic criteria.

2. Important demographic data (age and gender) are reported with mean (or median) and SDs (or range).

3. Healthy control subjects are evaluated to exclude psychiatric and medical illnesses and demographic data are reported.

4. Important clinical variables (e.g., medication status, illness duration and severity) are reported with mean (or median) and SDs (or range).

5. Sample size per group ＞10.

**Category 2: methodology and reporting**

6. Whole brain analysis is automated with no a priori regional selection.

7. Magnet strength at least 1.5T.

8. The acquisition and preprocessing techniques are clearly described so that they can be reproduced.

9. Coordinates reported in a standard space.

10. Significant results are reported after correction for multiple testing using a standard statistical procedure (AlphaSim, FDR, FWE or permutation- based methods).


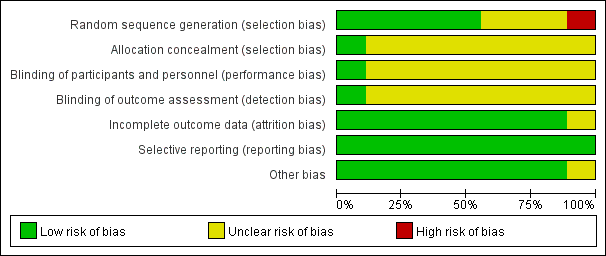


**Supplementary Figure 1.** Risk of bias graph


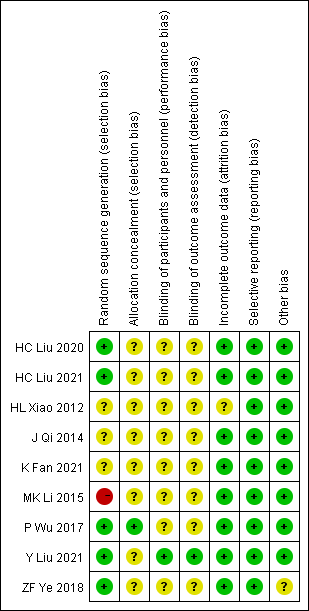


**Supplementary Figure 2.** Risk of bias summary
